# Predicting target proteins for drug candidate compounds based on drug-induced gene expression data in a chemical structure-independent manner

**DOI:** 10.1186/s12920-015-0158-1

**Published:** 2015-12-18

**Authors:** Yoshiyuki Hizukuri, Ryusuke Sawada, Yoshihiro Yamanishi

**Affiliations:** Faculty of Exploratory Technology, Asubio Pharma Co. Ltd., 6-4-3 Minatojima-Minamimachi, Chuo-ku, Kobe, Hyogo 650-0047 Japan; Division of System Cohort, Multi-Scale Research Center for Medical Science, Medical Institute of Bioregulation, Kyushu University, 3-1-1 Maidashi, Higashi-ku, Fukuoka, Fukuoka 812-8582 Japan; Institute for Advanced Study, Kyushu University, 6-10-1, Hakozaki, Higashi-ku, Fukuoka, Fukuoka 812-8581 Japan

**Keywords:** Drug target prediction, Drug-induced gene expression profiles, Transcriptome data, Machine learning

## Abstract

**Background:**

Phenotype-based high-throughput screening is a useful technique for identifying drug candidate compounds that have a desired phenotype. However, the molecular mechanisms of the hit compounds remain unknown, and substantial effort is required to identify the target proteins associated with the phenotype.

**Methods:**

In this study, we propose a new method to predict target proteins of drug candidate compounds based on drug-induced gene expression data in Connectivity Map and a machine learning classification technique, which we call the “transcriptomic approach.”

**Results:**

Unlike existing methods such as the chemogenomic approach, the transcriptomic approach enabled the prediction of target proteins without dependence on prior knowledge of compound chemical structures. The prediction accuracy of the chemogenomic approach was highly depended on compounds structure similarities in data sets. In contrast, the prediction accuracy of the transcriptomic approach was maintained at a sufficient level, even for benchmark data consisting of structurally diverse compounds.

**Conclusions:**

The transcriptomic approach reported here is expected to be a useful tool for structure-independent prediction of target proteins for drug candidate compounds.

## Background

Phenotype-based high-throughput screening (PHTS) is a useful technique for identifying drug candidate compounds that have a desired phenotype; thus, it is a prominent part of the drug development process [1]. However, when using PHTS alone, the underlying molecular mechanisms of hit compounds remain unknown, and considerable effort is usually required to identify the target proteins associated with the phenotype. Obtaining information on the mechanisms of drug actions (e.g., the primary target, off-targets, and active pathways) can help to optimize the structure of drug candidates and elucidate their potential side effects. Therefore, there is a strong incentive for researchers to efficiently predict the target proteins of drug candidate compounds.

Recently, a variety of *in silico* methods for compound target prediction have been proposed in the context of chemogenomics, where target prediction is based on compound structures and protein sequences as well as pre-existing knowledge from databases about known compound–protein interactions [2–7]. Chemogenomic methods work well when query compounds (e.g., drug candidate compounds) and the known target compounds in these databases share similar chemical structures. In contrast, when the chemical structures of these compounds share little similarity, chemogenomic methods are often ineffective. Recently, the use of information on the side effects of drugs has been proposed as an alternative method for target predictions [8–10]. Although side effect-based methods do not depend on the similarly of the compounds’ chemical structures, they are applicable only to those approved drugs for which detailed side effect profiles are available. Therefore, side effect-based methods cannot be applied to new drug candidate compounds (e.g., newly synthesized compounds) that are yet to have their side effects profiled.

Recent advances in transcriptome technologies (e.g., DNA-chips and RNA-seq) have enabled us to measure the expression profiles of all human genes at low cost, and several databases containing gene expression data have been constructed worldwide [11–13]. Connectivity Map (hereafter referred to as “CMap”) is a well-established database in which gene expression profiles for the chemical perturbations of 1,309 bioactive compounds in four cell lines are stored [14]. Broad Institute in the USA released CMap in 2006, and since then several studies have reported correlations between drug actions and the drug-induced gene expression patterns in the database [15–20]. In particular, the CMap resource has useful pharmaceutical applications, such as drug repositioning.

In this study, we propose a new method to predict target proteins of drug candidate compounds, termed the “transcriptomic approach,” which is based on drug-induced gene expression data in CMap with a machine learning classification technique. We compare the performance of the transcriptomic approach with that of the chemogenomic approach, which is based on chemical structures and protein sequences, and we show that the transcriptomic approach can predict target proteins independent of data on compound chemical structures. The prediction accuracy of the transcriptomic approach was maintained at a sufficient level, even for benchmark data consisting of structurally diverse compounds. Therefore, the transcriptomic approach is expected to be useful for predicting target proteins of drug candidate compounds in a chemical structure-independent manner.

## Methods

### Drug-induced gene expression data

CMap (build 02) is a collection of 6,100 gene expression profiles for 13,469 human genes from four cell lines (MCF7, HL60, PC3, and SKMEL5) treated with 1,309 bioactive small compounds. The CEL files of CMap were downloaded from the database website [21]. The CMap annotation file (cmap_instances_02.txt) indicates the distinct instance ID for each pair of treatment-control samples with experimental conditions (i.e., concentration, cell line, and batch). A filtering process was applied to this dataset as follows. First, MCF7 cell line instances were selected because MCF7 is the most frequently used of the four cell lines. Next, the instance with the highest concentration of treatment was selected when the same compounds were assigned different instances. The instance with a smaller batch ID value was selected if the instance with the same condition instance was present in different batches. Following this filtering process, 1,294 instances (i.e., compounds) were finally selected. MAS5 normalization was applied to all selected samples [22]. The GeneChip array (HG-U133A) has multiple probes assigned to one gene. The unique representative probe was selected by using the highest average rank based on the rank ordered matrix of expression changes between treatments and controls. The fold change score was calculated for each treatment against the corresponding control, and the base-2 logarithm was calculated. Finally, a 1,294 × 13,469 gene expression matrix (comprising 1,294 compounds in rows and 13,469 genes in columns) was constructed and denoted by X.

The gene expression similarities of compounds and of proteins (hereafter referred to as “compound expression similarities” and “protein expression similarities,” respectively) were evaluated by using Pearson’s correlation coefficients on the row and column profiles of the gene expression matrix, respectively. The expression profile of each compound is a real-valued feature vector, so we used Pearson’s correlation coefficient for "compound expression similarities", and the expression profile of each protein is a real-valued feature vector, so we used Pearson’s correlation coefficient for "protein expression similarities". In fact, Pearson’s correlation coefficient is a standard similarity measure in transcriptomic data analysis.

### Compound chemical structures and protein amino acid sequences

The compound structures used in CMap were obtained from ChemBank [23, 24]. The 2D frequency chemical descriptor of each compound was calculated by using the DragonX program [25], where the number of chemical substructures in the DragonX descriptor was 780. The chemical structure similarity scores of compound pairs were evaluated with generalized Jaccard coefficients. Protein amino acid sequences were downloaded from UniProt [26, 27]. The sequence similarity scores of protein pairs were calculated by using the Smith-Waterman algorithm in the EMBOSS water program (parameters: gap open penalty = 10.0, gap extension penalty = 0.5), and they were normalized via a cosine operation such that the maximal value was 1 and the minimal value was 0 [28, 29].

### Compound–target interactions

The information about compound–target interactions was obtained from DrugBank [30, 31] and ChEMBL [32, 33]. For the target information in ChEMBL, we used only target proteins labeled “active” in the comments section. In total, there were 4,870 unique compound–protein interactions in the merged dataset, with 756 compounds and 584 proteins involved in these interactions. We used all possible compound-protein pairs excluding positive examples as negative examples. Thus, the number of positive examples is 4870 and the number of negative examples is 436634. We used this dataset as the gold standard to evaluate the performance for compound target prediction. To investigate the validity of new predictions, we used experimental data from ChEMBL on compound–target interactions (e.g., binding affinity of <30 *μ* M in IC50), as well as independent information about compound–target interactions stored in KEGG [34], Matador [35], and PDSP Ki [36].

### Direct method for compound target prediction

The most straightforward method for compound target prediction involves directly using the fold change values of the associated compound–protein pairs in the gene expression matrix, X. The rationale for using this method is that drug-affected genes are highly variable in the observed gene expression. Thus, high scoring compound–protein pairs are predicted to be candidates for interacting compound–protein pairs. In this study, this method is referred to as the “direct method.”

We tested three possible scores for compound–protein pairs: i) X, ii) –X, and iii) abs (X). For the X score, the original fold change values of X are used, and higher scores correspond to up-regulated genes. For the –X score, the sign-inversed values of X are used, and higher scores correspond to down-regulated genes. For the abs (X) score, the absolute values of X are used, and higher scores correspond to up- or down-regulated genes. Compound-protein pairs with high scores in i) X, ii) –X, and iii) abs (X) are predicted to be candidates of interacting compound-protein pairs.

### Classification method for compound target prediction

Another method for compound target prediction involves comparing the gene expression profiles of query compounds with those of other compounds of known target proteins. For this method, the rationale is that drugs with similar gene expression patterns are likely to share common target proteins.

We attempted to solve the problem of compound target prediction by supervised classification, which consists of the following two steps. First, a classifier for discriminating interacting compound–protein pairs from the other pairs is learned based on partially known compound–protein interactions. Second, the learned classifier is applied to new compound–protein pairs, and high scoring compound–protein pairs are predicted to be candidates for interacting pairs. In this study, this method is referred to as the “classification method.” The related classification algorithms or their variants have also been used in the context of chemogenomics in several previous studies [2–7].

Given a training set of *n*_*x*_ × *n*_*y*_ compound–protein pairs (x_*i*_, *y*_*j*_) (*i* = 1 ⋯ *n*_*x*_; *j* = 1, ⋯ *n*_*y*_), *f*(x ', y ') should be estimated to predict whether a compound **x**’ interacts with a protein y’. Among the pair classification algorithms, we used pairwise kernel regression (PKR) [9] because of its computational efficiency. We define a similarity function *k*_*compound*_ for compounds and a similarity function *k*_*protein*_ for proteins; thus, a statistical model of PKR for a given compound–protein pair (x’,y’) is defined as follows:

$$ f\left(\mathrm{x}\hbox{'},\mathrm{y}\hbox{'}\right)={\displaystyle \sum_{i=1}^{n_x}{\displaystyle \sum_{j=1}^{n_y}{\beta}_{ij}{k}_{compound}\left({\mathrm{x}}_j,\mathrm{x}\hbox{'}\right)}{k}_{protein}\left({\mathrm{y}}_j,\mathrm{y}\hbox{'}\right)} $$

*β*_*ij*_ is a weight parameter to be optimized based on the training set. In practice, high-scoring compound–protein pairs are predicted as the interacting pairs. The inputs of the PKR model are similarity scores for both compound pairs and protein pairs. Therefore, the performance depends heavily on the similarities of compounds and proteins.

In the transcriptomic approach, we used compound expression similarity (Pearson’s correlation) for “compound similarity” and protein expression similarity (Pearson’s correlation) for “protein similarity”. In the chemogenomic approach, we used chemical structure similarity (generalized Jaccard coefficient) for “compound similarity” and protein sequence similarity (normalized Smith-Waterman score) for “protein similarity”. In the integrative approach, we used the average of compound expression similarity and compound structure similarity for “compound similarity” and the average of protein expression similarity and protein sequence similarity for “protein similarity”.

### Predictive performance measures

We evaluated the predictive performance by using the receiver operating characteristic (ROC) curve and Precision-Recall (PR) curve, which are plots of true positive rates against false positive rates based on various thresholds (ROC) and precision (positive predictive value) against recall (sensitivity) based on various thresholds for the prediction score (PR), respectively. We computed the area under the ROC curve (i.e., the AUC score), where 1 is returned for a perfect inference and 0.5 is returned for a random inference, and the area under the PR curve (i.e., AUPR score), where 1 is returned for a perfect inference and the ratio of positive examples against all samples in the gold standard data is returned for a random inference. We used the ROCR package in the R language. In the program, many threshold values (e.g., 1, 0.9, 0.8, 0.7, ….,–0.8,–0.9, -1) were prepared for the predictions scores, the true positive rates against the false positive rates at each threshold value were plotted, and the ROC curve was drawn by connecting the dots. The AUC and AUPR scores were computed for each compound, and the average scores were used to summarize the results.

We also evaluated the performance of top ranked predictions by determining whether known target proteins appeared in the top 10 or top 50 high-scoring predictions. The accuracy of top ranked predictions is important in practice because experimental validation will be preferentially conducted on high-confidence predictions rather than low-confidence predictions. We computed the ratio of the number of correctly predicted compounds with at least one known target protein in the top 10 or top 50 against the number of all compounds of known targets in the gold standard data. We denote the ratio scores for top 10 and top 50 as “Top10 ratio” and “Top50 ratio,” respectively. AUC/AUPR represents the global accuracy of all compounds in each benchmark dataset, while Top10/Top50 represents the local accuracy of top compounds in each benchmark dataset. Note that the prediction accuracy scores were evaluated from different viewpoints.

### Experimental protocol for the classification method

The classification method requires supervised learning with pre-existing knowledge about known compound–protein interactions. To evaluate the predictive performance of the classification method, we performed a cross-validation experiment using the gold standard compound–protein interaction data. This experiment was designed to simulate the practical situation in which researchers are required to predict the potential target proteins of new drug candidate compounds.

We performed the following 5-fold cross-validation. (i) We randomly split compounds into five compound subsets. (ii) We took each compound subset as test compounds, and constructed a test set of compound–protein pairs. (iii) We took the remaining compound subsets as training compounds, and constructed a training set of compound–protein pairs. (iv) We trained a classifier using the training set, and applied it to the test set. (v) We computed the prediction scores for compound–protein pairs in the test set, and evaluated the prediction accuracy over the 5-fold process. It should be noted that only compounds were split into the training and test sets, and the list of proteins was common across both sets.

### Construction of several benchmark datasets containing compounds with diverse chemical structures

The gold standard dataset contained many drugs that are structurally almost identical because some of them are derivatives of the same lead compound. If these identical drugs were divided into a training set and a test set, the prediction in the cross-validation experiment would be trivial. To avoid overestimation of the prediction accuracy, we therefore performed filtering of similar drugs based on their chemical structures. Hence, we used only diverse drugs that were structurally different to some extent.

The process proceeded as follows. First, we calculated the generalized Jaccard coefficient (Tanimoto coefficient for real-valued feature vectors) of chemical descriptors for all compounds. Second, we identified the compounds that shared high Jaccard coefficients and selected one representative compound based on a threshold. Third, we constructed a set of representative compounds that shared low Jaccard coefficients. Finally, we prepared seven sets of benchmark data consisting of representative drugs by gradually varying the chemical similarity threshold (e.g., from 0.4 to 1.0 by increments 0.1) on the dendrogram. When the threshold is <0.4, the number of drug clusters is very small; thus, it was not possible to test thresholds of 0.1–0.3 in the 5-fold cross-validation.

## Results

### Drug-induced gene expressions are not correlated with compound structures or target protein sequences

**Fig. 1:**
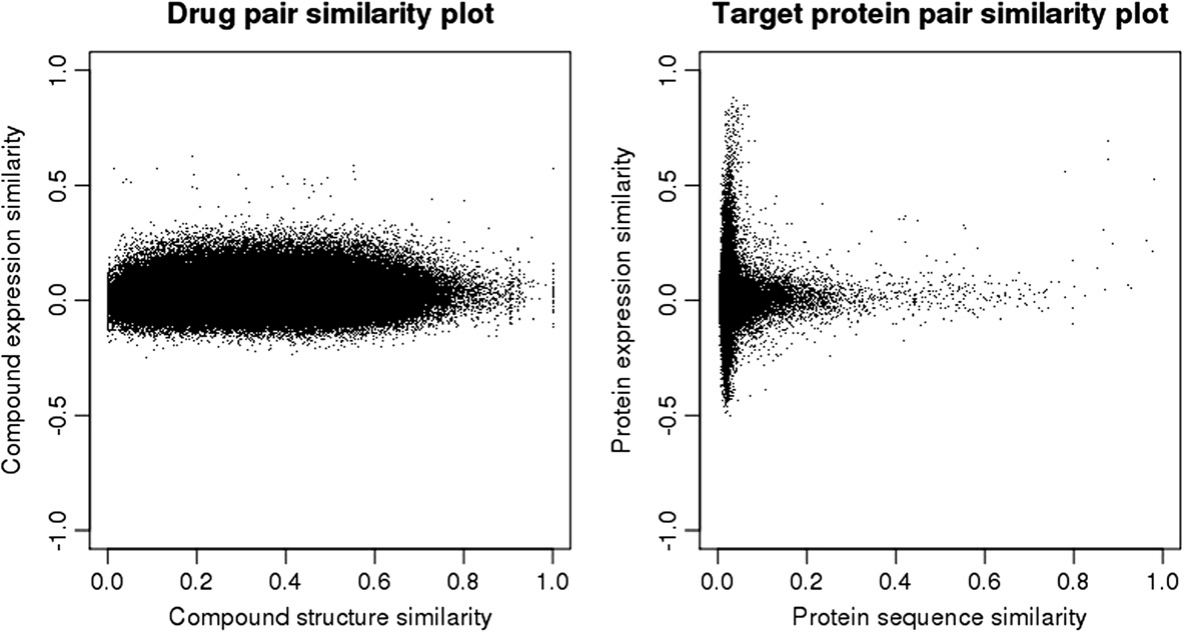
Distributions of drug similarities and protein similarities. The left and right panels show scatter plots of drug expression similarities against drug structure similarities and protein expression similarities against protein sequence similarities, respectively

As a preliminary analysis, we examined the correlation between drug-induced gene expressions and compound chemical structures or target protein sequences. We also analyzed the distributions of compound expression similarities, compound structure similarities, protein expression similarities, and protein sequence similarities. Figure 1 shows scatter plots of compound and protein expression similarity scores against compound structure and protein sequence similarity scores, respectively. Compound expression similarities were not correlated with compound chemical structure similarities, nor were protein expression similarities correlated with protein sequence similarities. These results imply that drug-induced gene expression profiles can provide characteristic information that is different from the information provided by compound chemical structures and protein amino acid sequences.

### Comparison of different scoring schemes for drug-induced gene expression

**Fig. 2:**
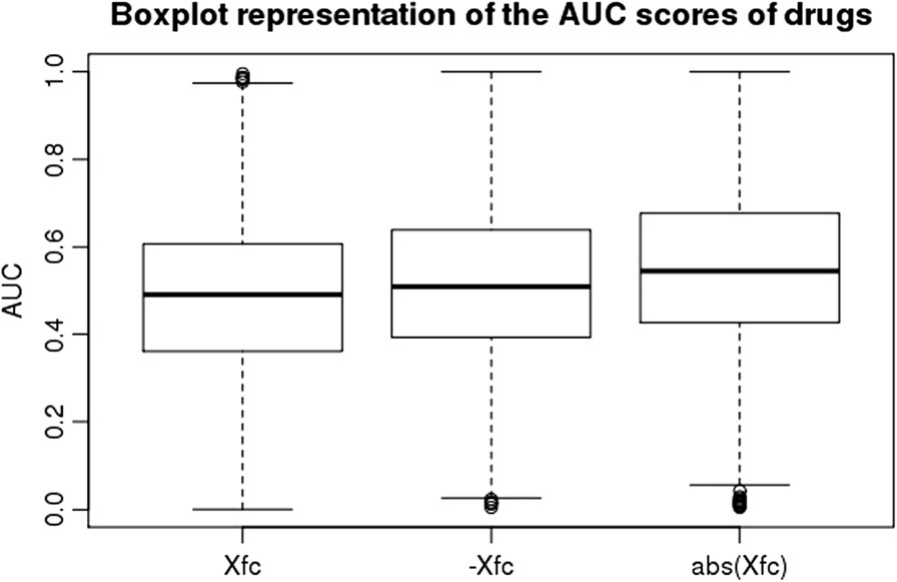
Comparison of gene expression values. Boxplots illustrating the AUC scores of drugs based on X, −X, and abs(X) scores

We tested the direct method with three scoring schemes for drug-induced gene expression values, namely X, –X, and, abs(X), and evaluated their correspondences to known compound–protein interactions using the gold standard compound–protein interaction data. Based on the distributions of AUC scores for compounds when using X, –X, and, abs(X), the abs(X) score showed the superior performance, with X and –X resulting in almost random inference (Fig. 2). Negative values in X correspond to the down-regulation of genes by drugs (i.e., inhibitors), and positive values in X correspond to up-regulation (i.e., activators). Therefore, this result suggests that the abs(X) scores reflect drug-induced gene expression variability involving both up- and down-regulated genes. Consequently, we used the abs(X) scores as drug-induced gene expression profiles in the following analysis.

### The use of pre-existing knowledge on known compound–protein interactions improves predictive performance

**Fig. 3:**
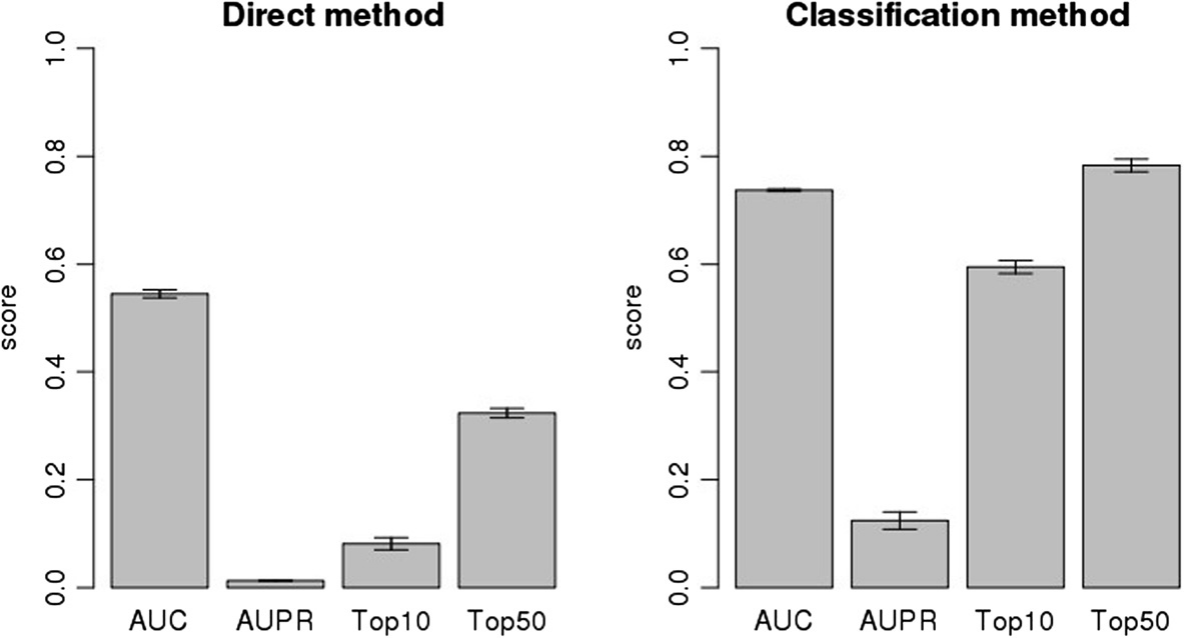
Comparison of the direct and classification methods. The left and right panels show the performance of the direct method and the classification method, respectively. Average AUC scores, average AUPR scores, the Top10 ratio, and the Top50 ratio are illustrated

We tested the classification method with compound expression similarities and protein expression similarities as the inputs of the PKR model, and we evaluated its performance in compound target prediction by performing 5-fold cross-validation experiments on the gold standard data. In addition, we compared the predictive performance of the classification method with that of the direct method. Based on a comparison of the AUC and AUPR scores, and Top10 and Top50 ratios, from the direct method with those obtained from the classification method (Fig. 3), the classification method exhibited superior performance for all measures. This result suggests that using pre-existing knowledge of known compound–protein interactions can improve the predictive performance of the method; thus, a supervised learning framework is encouraged for compound target prediction in practice.

### The transcriptomic approach is useful for structure-independent prediction of compound targets

**Fig. 4:**
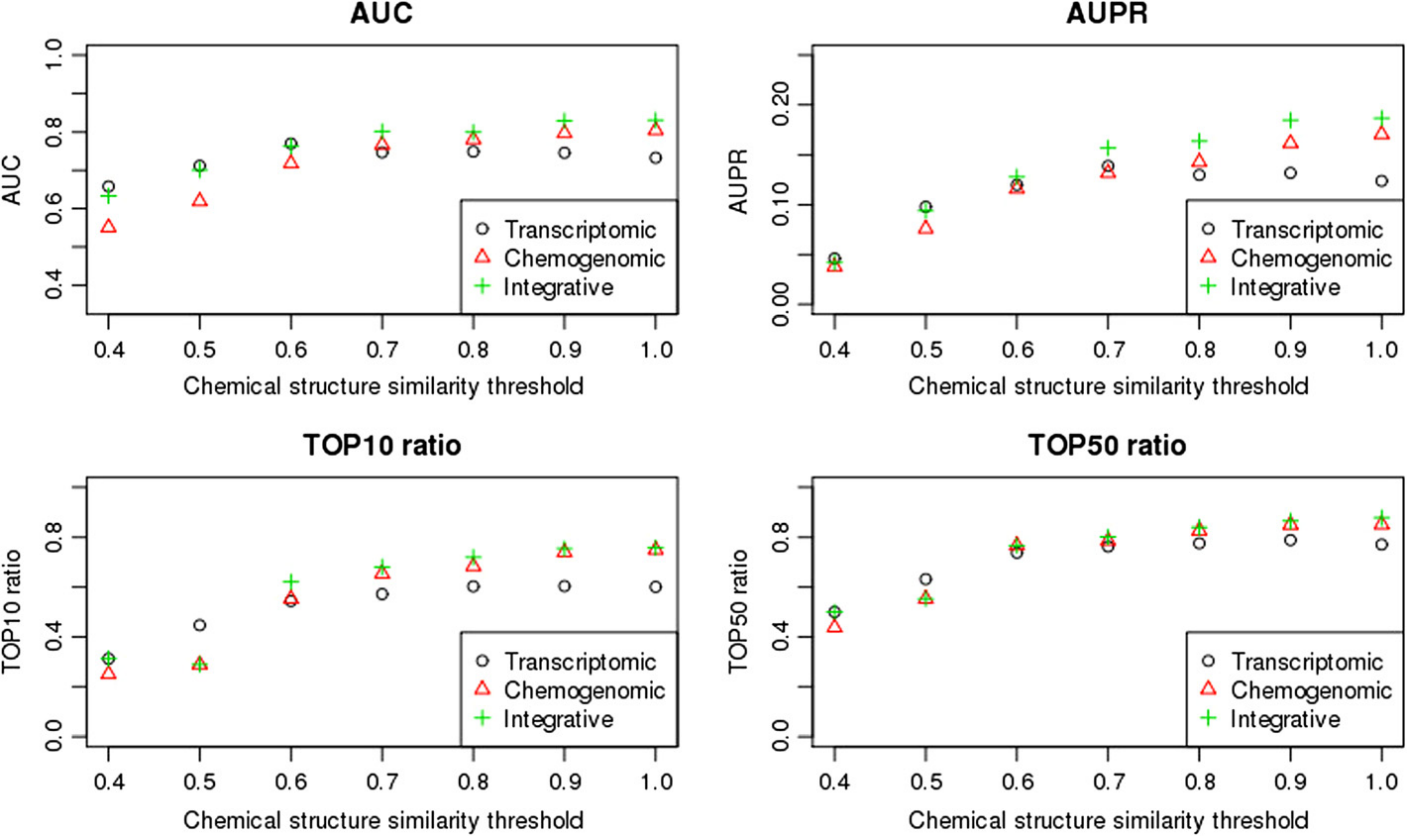
Performance of the transcriptomic approach, chemogenomic approach, and the integration of the two approaches. The performance of each approach is based on benchmark datasets containing compounds with different chemical diversities. The upper left panel shows the average AUC scores, the upper right panel shows the average AUPR scores, the bottom left panel shows the Top10 ratio, and the bottom right panel shows the Top50 ratio

We compared the performance of the transcriptomic approach with that of chemogenomic approach (note that the transcriptomic approach corresponds to the classification method with compound and protein expression similarity, whereas the chemogenomic approach corresponds to the classification method with compound structure and protein sequence similarity). Figure 4 shows the AUC and AUPR scores, and the Top10 and Top50 ratios, for the transcriptomic and chemogenomic approaches, with results based on various benchmark datasets derived from different chemical structure similarity thresholds. The chemogenomic approach worked well for benchmark data consisting of many structurally similar compounds (e.g., those with chemical structure similarity thresholds of 1 or 0.9), but it worked poorly for structurally diverse compounds (e.g., those with similarity thresholds of 0.4 or 0.5). This is perhaps explained by the fact that many compounds correspond to drug derivatives optimized from the same compound lead; thus, prediction with chemical structures is relatively simple in a cross-validation experiment when the test and training compounds have highly similar chemical structures. Conversely, the transcriptomic approach maintained high prediction accuracy regardless of the benchmark dataset, which implies that this approach can predict compound targets independent of compound chemical structures. These results suggest that the transcriptomic approach is useful for predicting unknown compound targets that are not expected from compound chemical structures.

We also investigated the effects of integrating the transcriptomic approach with the chemogenomic approach, which we called the “integrative approach.” Note that the input in the integrative approach is the average of the compound expression similarity and compound structure similarity. As shown in Fig. 4, the integrative approach produced the superior performance in most cases, but showed similar results to the transcriptomic approach when benchmark data consisted of structurally diverse compounds. One explanation about the observation would be that the performance of the integrative approach was deteriorated by the averaging with the uninformative compound structure similarity. This suggests that the integration of transcriptome data with chemical information is most useful when query compounds share a certain amount of chemical structure similarity with compounds in the training set.

### Novel target prediction for compounds in CMap

Finally, we conducted a comprehensive prediction of unknown compound–protein interactions involving 1,294 compounds and 13,469 proteins-coding genes in CMap. We trained a predictive model using all known compound–protein interactions in the gold standard data, and predicted unknown compound–protein interactions. Consequently, by using the transcriptomic, chemogenomic, and integrative approaches, we predicted potential off-targets for 756 compounds of the known targets (compounds present in the gold standard data) and potential target profiles for 538 uncharacterized compounds of unknown targets (compounds absent from the gold standard data).

**Fig. 5:**
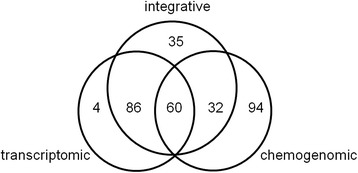
Venn diagram of correctly predicted compound–protein pairs in the transcriptomic, chemogenomic and integrative approaches. The three circles represent the correctly predicted compound–protein pairs from the transcriptomic approach, chemogenomic approach, and integrative approach, respectively

We investigated the validity of the top 1,000 high-scoring predictions by using independent resources on compound–protein interactions and the latest information in KEGG, DrugBank, Matador, ChEMBL, and PDSP Ki. As a result, we confirmed the validity of 150 predictions from the transcriptomic approach and 186 predictions from the chemogenomic approach and 213 predictions from integrative approach. Figure 5 shows a Venn diagram with the overlap of only 60 common correctly predicted compound–protein interaction pairs between the two approaches. Thus, there is apparently a large difference between the compound–protein pairs that are correctly predicted by each approach.

**Fig. 6:**
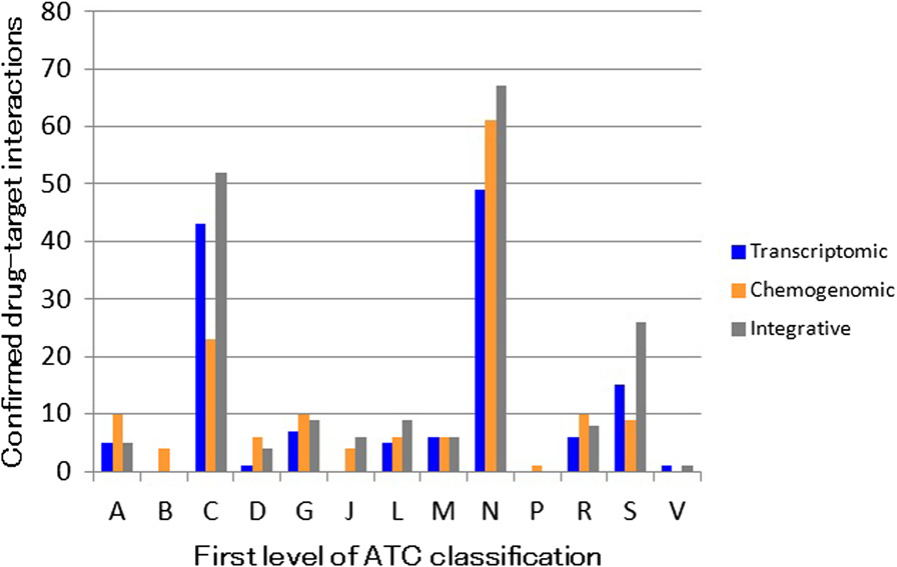
Anatomical Therapeutic Chemical (ATC) classification of compounds in correctly predicted compound–protein pairs. Compounds are labeled by the first level ATC code and the frequency of each class is shown. The blue, orange, and gray bars indicate the number of compounds predicted by the transcriptomic approach, chemogenomic approach, and integrative approach, respectively

Figure 6 shows the frequency of ATC classification (Anatomical Therapeutic Chemical Classification System) for the compounds in correctly predicted compound–protein interaction pairs. In addition, Table 1 shows a list of target proteins found in the correctly predicted compound–protein interaction pairs from the transcriptomic, chemogenomic, and integrative approaches. Most proteins in the high scoring prediction pairs from the transcriptomic approach were those known to be associated with the mechanism of drug actions. For example, the ATC classification showed that the compounds corresponded largely to drugs for the cardiovascular system (those with the first level code “C” in Fig. 6), and the associated proteins corresponded to neurotransmitter receptors (e.g., ADRB3 in the GPCR family). This implies that neurotransmitter antagonists have characteristic gene expression profiles, which is a finding also supported by previous work [19].

**Table 1:** A list of proteins from the correctly predicted compound–protein pairs

Transcriptomic		Chemogenomic		Integrative	
GeneName	Number	GeneName	Number	GeneName	Number
ADRB3	15	CYP2C9	22	ADRB3	17
CA1	5	CYP2C19	16	ADRA1D	7
DRD5	5	CYP1A2	14	TOP2B	7
HTR2B	5	CHRM4	6	CA1	6
ADRA1B	5	CHRM5	6	DRD5	6
CA7	4	CHRM3	5	ADRA2C	6
CACNG1	4	ESR2	5	CACNG1	5
CHRM4	4	DRD1	4	HTR2B	5
BCHE	4	DRD2	4	ADRA1B	5
SLC12A2	4	CA7	4	BCHE	5
CA12	4	HTR2C	4	CA12	5
ADRA2C	4	CA1	3	HTR2C	4
HTR2C	3	HRH1	3	CHRM5	4
CHRM5	3	HSD17B10	3	CA7	4
ADRA1D	3	HTR1B	3	DRD3	4
CACNA1I	3	ADRA1A	3	CHRM4	4
CA9	3	ADRA2A	3	CA3	4
SLC12A3	2	THPO	3	SLC12A2	4
CACNA1S	2	ALOX15	3	CA9	4
ABCC8	2	ADRA1D	3	CA14	4
HTR1B	2	CA3	3	CA4	4
DRD3	2	BCHE	3	HTR1A	3
HDAC2	2	SLC12A3	2	ESR1	3
DRD4	2	ESR1	2	CACNA1H	3
HTR1A	2	MAPT	2	CACNA1I	3
HTR7	2	PTGS1	2	SMN1/SMN2	3
ESR1	2	PTGS2	2	ESR2	3
ALOX12	2	ALDH1A1	2	CYP1A2	3
CA3	2	ABCC8	2	SLC12A3	2
CA4	2	DRD5	2	CACNA1S	2
CA14	2	KCNH2	2	ABCC8	2
GABRA3	2	CACNG1	2	HTR1B	2
GABRA2	2	HTR1A	2	HDAC2	2
SMN1/SMN2	2	ADRB3	2	DRD4	2
GABRA6	2	MTOR	2	PTGS1	2
GABRA4	2	SLC12A2	2	HTR7	2
PTGS1	2			ALOX12	2
ADRA1A	2			SLC6A3	2
				RPS6KA3	2
				DRD1	2
				CHRM3	2
				GABRA3	2
				GABRA2	2
				GABRA4	2
				GABRA6	2
				PKM	2
				CYP2C9	2
				CYP2C19	2

Proteins in correctly predicted compound–protein pairs, which were obtained by using the transcriptomic approach, chemogenomic approach, and integrative approach, are listed. The list shows proteins that were correctly predicted multiple times

In contrast, more than 50 prediction pairs from the chemogenomic approach were proteins associated with metabolism of drugs. For example, the top ranked target proteins corresponded mainly to drug metabolizing enzymes (e.g., CYP2C9 in the cytochrome P450 family), which are known to have low substrate specificity and to bind many compounds with ubiquitous properties such as large dipole, negative charge, oxygen-richness, and aromatic rings [37, 38]. These results suggest that the high scoring predictions from the chemogenomic approach are likely to indicate target proteins that are not associated with the mechanisms of drug action.

## Discussion

In this study, we examined the performance of computational methods for CMap-based compound target prediction. We observed that the direct method worked poorly, and was only slightly better than random inference. This suggests that chemically perturbed genes do not always correspond to target proteins of a query compound and the direct method is therefore not useful in practice. In contrast, the classification method performed well, which indicates that comparing gene expression profiles among a query compound and other compounds of known targets is more useful than simply selecting the regulated genes in the gene expression profile of the query compound.

The previously developed methods for predicting drug/compound targets can be mainly categorized into gene expression-based methods such as CMap and chemical structure-based methods such as the chemogenomic approach. However, to the best of our knowledge, the relationship between the two types of methods has not previously been investigated; therefore, this study was the first to systematically compare the usefulness of gene expression and chemical structure information in the context of compound target prediction (with the same benchmark data tested under unbiased experimental conditions). Our results showed little correlations between gene expression and chemical structure similarities, which suggests that the two resources are complementary.

We also observed that the chemogenomic approach worked poorly when test compounds did not share sufficient similarity in chemical structure, whereas the transcriptomic approach maintained prediction accuracy even without this similarity. Thus, the performance of the transcriptomic approach is apparently independent of compound chemical structures, and this property has practical importance for predicting the targets of compounds that have novel chemical structures.

We observed that the correctly predicted compound–protein interaction pairs with high confidence scores differed depending on the approach used. For example, the top predictions from the chemogenomic approach contained low substrate-specific proteins (e.g., enzymes in the cytochrome P450 family). This might be because a variety of compounds that interact with such enzymes were included in the training data, and such predictions therefore may have arisen from the dependency on various chemical structures of the enzyme ligands. This property might also have contributed to the observation that the predictions from the chemogenomic approach were more accurate than those of the transcriptomic approach in the cross-validation experiment with full benchmark data derived from a chemical similarity threshold of 1. In contrast to those of the chemogenomic approach, the top predictions from the transcriptomic approach did not contain low substrate-specific proteins, but instead contained many proteins associated with the mechanism of drug actions (e.g., adrenergic receptors such as ADRB3 and ADRA1B). This implies that the transcriptomic approach is more likely to predict target proteins that affect the expression of genes at downstream pathways rather than predict drug-metabolizing enzymes that have no effect on gene expression. Because the transcriptomic and chemogenomic approaches have different properties in terms of compound target prediction, care should be taken to choose the appropriate approach depending on the research objective.

The majority methods for drug/compound target prediction are based on chemical structure, including the chemogenomic approach. One reason for its popularity is the huge number of compound structures that can be easily obtained at a low cost from the many molecular databases such as DrugBank and ChEMBL. Conversely, considerable experimental costs are required to obtain drug-induced gene expression profiles. The CMap project has changed this situation somewhat by providing a large number of drug-induced gene expression profiles, and has thereby enabled the comparison of compounds in terms of their gene expression [14]. Because recent technological advances such as the development of next generation sequencers provide the potential to measure more gene expression profiles at lower cost, CMap and similar projects will be able to expand the transcriptomic space of possible compounds in the near future [39–43]. Consequently, the transcriptomic approach developed here will be a useful tool for exploring the targets of drug candidate compounds with novel chemical structures.

## Conclusions

In this paper, we proposed a new method to predict target proteins of drug candidate compounds based on drug-induced gene expression data from CMap and a machine learning classification technique. We compared the performance of this method, termed the transcriptomic approach, with that of the chemogenomic approach, and we found that it does not depend on compound chemical structures and is suitable for predicting target proteins that cannot be expected from analyzing such structures. To the best of our knowledge, this is the first study to report the relationship between a transcriptomic approach and a chemogenomic approach to target prediction. In practice, the novel transcriptomic approach described here is expected to be particularly useful for predicting target proteins for drug candidate compounds in a chemical structure-independent manner.
